# Analysis of the Mechanical and Biofilm-Inhibitory Antimicrobial Properties of a Dental Tissue Conditioner Incorporating *Ocimum Gratissimum* Essential Oil: An In Vitro Study

**DOI:** 10.1155/ijod/9994172

**Published:** 2025-06-29

**Authors:** Tri Minh Doan, Chau Tran Bao Vu, Phuong Thi Luc Truong, Van-Khoa Pham, Natdhanai Chotprasert

**Affiliations:** ^1^Department of Prosthodontics, Faculty of Dentistry, University of Medicine and Pharmacy at Ho Chi Minh City, Ho Chi Minh City, Vietnam; ^2^Faculty of Odonto-Stomatology, University of Health Sciences, Vietnam National University Ho Chi Minh City, Ho Chi Minh City, Vietnam; ^3^Faculty of Dentistry, University of Medicine and Pharmacy at Ho Chi Minh City, Ho Chi Minh City, Vietnam; ^4^Department of Operative Dentistry and Endodontics, Faculty of Dentistry, University of Medicine and Pharmacy at Ho Chi Minh City, Ho Chi Minh City, Vietnam; ^5^Faculty of Dentistry, Mahidol University, Bangkok, Thailand

**Keywords:** antimicrobial properties, dental tissue conditioner, *Ocimum gratissimum* essential oil, tensile bond strength

## Abstract

**Objectives:** This study aimed to investigate the tensile bond strength (TBS) and biofilm-inhibitory antimicrobial properties of tissue conditioners combined with *Ocimum gratissimum* essential oil (EO) at varying concentrations.

**Materials and Methods:** The original tissue conditioner was used as the control, while the experimental groups consisted of tissue conditioners incorporating *O. gratissimum* EO at concentrations of 1% and 2% (*v*/*v*) in the liquid component. The TBS between the tissue conditioner and denture base acrylic resin was measured using a universal testing machine. To evaluate biofilm-inhibitory antimicrobial properties, cylindrical specimens infused with EO were prepared and incubated with *Candida albicans* and *Streptococcus mutans*. The crystal violet assay was utilized to quantify microbial biofilm formation.

**Results:** The incorporation of *O. gratissimum* EO into tissue conditioners significantly increased the TBS on day 1 (*p*  < 0.05) but showed no effect by day 7 post-polymerization (*p*  > 0.05). Additionally, tissue conditioners containing 1% EO exhibited biofilm-inhibitory antimicrobial properties on day 1 (*p*  < 0.05), whereas those with 2% EO demonstrated biofilm-inhibitory antimicrobial activity on both days 1 and 3 postinoculation (*p*  < 0.05). By day 5 and 7, EO-infused tissue conditioners no longer exhibited biofilm-inhibitory antimicrobial properties (*p*  > 0.05).

**Conclusion:** Tissue conditioners infused with *O. gratissimum* EO effectively reduced the formation of biofilms by *C. albicans* and *S. mutans* in a dose–dependent manner on days 1 and 3. As tissue conditioners are typically replaced every 3–7 days, *O. gratissimum* EO can be incorporated as an additive to lower the formation of biofilms by *C. albicans and S. mutans* without compromising the TBS of the tissue conditioner to denture base acrylic resin.

## 1. Introduction

The field of maxillofacial prosthetics has advanced significantly, greatly enhancing the well-being and life quality for patients with maxillofacial defects following surgery [1]. Maxillofacial prostheses benefit individuals with congenital defects, injuries from traffic accidents, or workplace incidents, and particularly those recovering from cancer surgery by restoring essential functions, such as chewing, speech, and esthetics [1–3]. These interventions ultimately improve patients' overall quality of life [1–3]. Typically, patients receive a transitional prosthesis made from acrylic denture base resin and short-term soft liners immediately after surgery [2]. This prosthesis helps restore functions while alleviating the psychological impact of visible defects [2, 4]. Additionally, it minimizes the risk of infection and promotes healing in preparation for the final, definitive prosthesis [2, 4].

Microbial colonization on the surface of prosthetic devices in the oral cavity poses risks of infection and can hinder the wound healing process [5]. Microorganisms adhere to the prosthetic surface and penetrate its material through pores, cracks, and structural defects [5]. The development of local or systemic infections depends on the patient's general well-being and the pathogenicity of the microorganisms involved [6]. For patients undergoing surgery to remove maxillary tumors, subsequent radiation therapy and chemotherapy may induce temporary immunodeficiency [6]. This condition increases the risk of opportunistic infections, particularly oral mucositis, which is commonly associated with *Candida albicans* [7]. Consequently, there is a pressing need for clinicians to mitigate the risks of local infections and systemic complications by improving prosthetic materials or incorporating additives to restrain the invasion and growth of harmful microorganisms [8].

Tissue conditioners are commonly employed as temporary relines to reduce the transfer of masticatory loads and ensure the even distribution of external stresses on the mucosa of the supporting structure [9]. These liners have been enhanced with antifungal drugs as additives to treat patients infected with *Candida* spp. [10]. However, these drugs have not been shown to effectively kill microorganisms that interact with fungi within biofilms [10]. In this context, *Streptococcus mutans*, a prevalent microorganism in the oral microbiota, has been reported to interact with *C. albicans*, thereby increasing the virulence of the biofilm [11]. Studies investigating the incorporation of other antimicrobial agents, such as metal oxide powders, silver nanoparticles, and plant essential oils (EOs), into tissue conditioners have demonstrated effectiveness against both fungi and bacteria [12–14].

The essential oil (EO) of *Ocimum gratissimum* is renowned for its anti-inflammatory and antimicrobial properties, demonstrating effectiveness against both bacteria and fungi [15, 16]. This study aimed to evaluate the tensile bond strength (TBS) of tissue conditioners modified with *O. gratissimum* EO at various concentrations and to assess their biofilm-inhibitory antimicrobial efficacy against *C. albicans* and *S. mutans*. The null hypothesis proposed that there would be no significant differences in TBS or biofilm-inhibitory antimicrobial properties between the original tissue conditioners and those incorporated with different concentrations of *O. gratissimum* EO.

## 2. Methods

### 2.1. Investigation of TBS Between Tissue Conditioner and Denture Base Acrylic Resin

Tissue conditioners (*Coe-Comfort*, GC America Inc., Alsip, IL, USA) without any additives was prepared as per the manufacturer's instructions and marked as the control group (*n* = 5). In comparison, tissue conditioners combined with 1% and 2% (*v*/*v*) *O. gratissimum* EO (EO; Vietnam Essential Oil., Hanoi, Vietnam) in the liquid component were classified as test groups (*n* = 5 per group).

For the TBS testing, samples were fabricated using a “sandwich” design, where a tissue conditioner sheet was positioned between two polymethyl methacrylate (PMMA) sheets (*ProBase Hot*, Ivoclar Vivadent AG, Liechtenstein). First, acrylic resin denture base sheets measuring 25 mm × 25 mm × 3 mm were prepared as per the manufacturer's guidelines. These sheets were formed using the same mold, with the mold lid ensuring a smooth, polished surface. After complete polymerization, any excess acrylic that had overflowed from the mold was trimmed and the surfaces of the acrylic sheets were polished using P500 grinding paper.

In the next step, *O. gratissimum* EO was added to the liquid component of the tissue conditioner in the ratios specified in Table 1, prior to mixing with the powder component. While the mixture remained in a liquid state, it was poured into the middle layer of the press mold, which already had PMMA sheets positioned on the outer layers. Pressure was then applied to form the sample according to the design, resulting in a tissue conditioner sheet measuring 25 mm × 25 mm × 2 mm. Excess resin overflowing from the mold was trimmed after 8 min of polymerization, and the sample was further compressed for 1 h before being placed in an artificial saliva solution (*Saliva Orthana*, Nycomed, Little Chalfont, UK) and stored at 37°C.

The TBS test was conducted on days 1 and 7 following the incubation of the samples. The specimens were tested using a universal testing machine (*Lloyd 30 k*, Lloyd Instruments Ltd., Fareham, UK) at a displacement rate of 5 mm/min. The load (*N*) at the point of debonding was recorded, and the TBS, expressed in MPa, was calculated by dividing the debonding load by the surface area of the specimen [17].

### 2.2. Biofilm Formation Assays

Tissue conditioner disks incorporating *O. gratissimum* EO at three concentration levels (0% [control], 1% *v*/*v* [Group 1], and 2% *v*/*v* [Group 2]) were prepared as described above and placed into 24-well plates (Corning, St. Louis, MO, USA). Before exposure to microorganisms, all polymerized samples were sterilized using continuous UV light treatment for 60 min.

Standard strains of *S. mutans* (ATCC 25175) and *C. albicans* (ATCC 10231) were cultured on Brain Heart Infusion (BHI) and Sabouraud Dextrose Agar (SDA), respectively, and incubated at 37°C for 24 h. One colony of each strain was subsequently introduced into 20 mL of broth medium and incubated overnight at 37°C in an orbital shaker under aerobic conditions. The microbial cells were then collected and rinsed two times using sterile phosphate buffered saline (PBS). *S. mutans* and *C. albicans* were resuspended in Mueller–Hinton Broth (MHB) and RPMI-1640, respectively, with cell concentrations adjusted to 1.0 × 10^6^ cells/mL after being counted using a hemocytometer [12].

A volume of 500 μL of the prepared microbial strain suspensions was introduced onto the tissue conditioner disks containing *O. gratissimum* EO. Incubation of the disks was carried out at 37°C for durations of 1, 3, 5, and 7 days (*n* = 3 per group). Following the incubation period, the specimens were rinsed three times with PBS to remove unattached microorganisms. They were then stained with 200 μL of a 1% crystal violet solution for 30 min at room temperature. Excess dye was removed by washing the specimens at least three times with PBS. Following 24 h of drying, the biofilm was extracted using absolute alcohol for 30 min. The quantity of biofilm was then assessed by measuring the optical density (OD) at 595 nm.

### 2.3. Statistical Analysis

Data analysis was conducted using SPSS 23.0 (SPSS, Chicago, IL). The Shapiro–Wilk test was applied to evaluate the normality of the data distribution. One-way ANOVA, followed by the post hoc Tukey HSD test, was utilized to assess the differences in TBS of tissue conditioners containing *O. gratissimum* EO on days 1 and 7 post-polymerization, as well as the variations in OD of *C. albicans* and *S. mutans* biofilms formed on tissue conditioners.

## 3. Results

**Figure 1:**
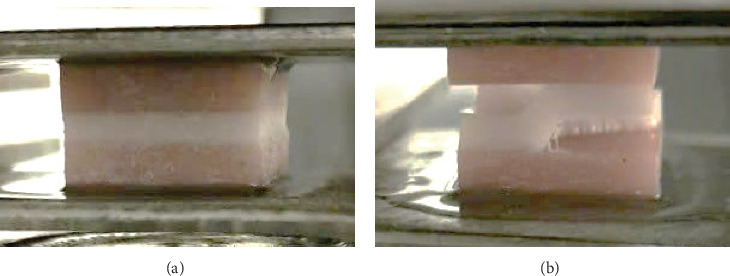
Specimen assembly for the tensile bond strength test. (A) Specimen before the load application. (B) Specimen with two adhesive surfaces delaminated between the tissue conditioner and the denture base acrylic resin after the load application.

**Figure 2:**
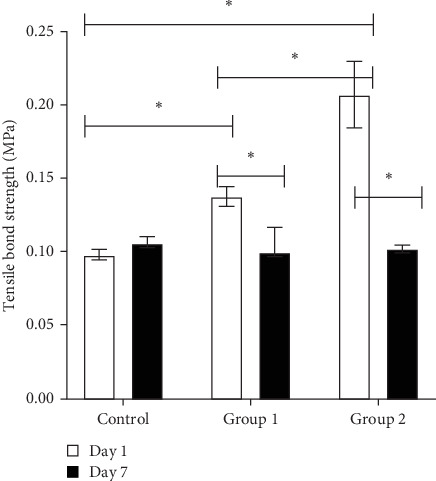
Tensile bond strength for each tissue conditioner group on day 1 and day 7 after polymerization, *⁣*^*∗*^*p* < 0.05 indicates statistical significance.

The TBS test revealed that all samples delaminated at one or two adhesive interfaces between the tissue conditioner and the acrylic resin denture base, with the tissue conditioner remaining free of fractures (Figure 1). On day 1, the TBS of tissue conditioners containing 1% EO (Group 1) and 2% EO (Group 2) increased significantly compared to the control group (0% EO) (*p*  < 0.05). However, after 7 days, no significant differences were noted among the three study groups (*p*  > 0.05). Within-group comparisons indicated that the TBS of the control group (0% EO) did not change significantly over the 7-day period (*p*  > 0.05). In contrast, the TBS of Group 1 and Group 2 decreased significantly from day 1 to day 7 (*p*  < 0.05) (Figure 2).

**Figure 3:**
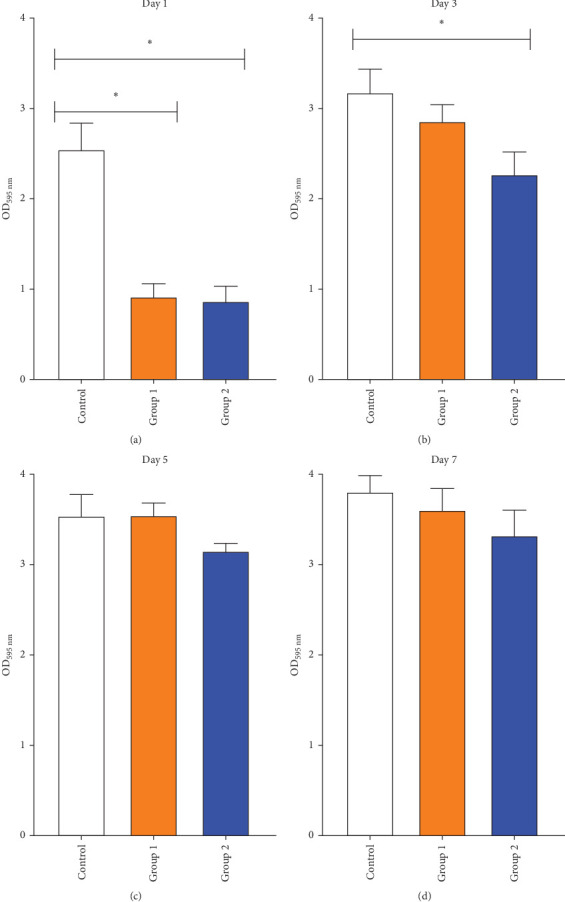
Inhibitory effect of tissue conditioners containing *O. gratissimum* EO on *C. albicans* biofilm formation at four time points postincubation. (A) Day 1; (B) Day 3; (C) Day 5; (D) Day 7, *⁣*^*∗*^*p* < 0.05 indicates statistical significance.

The quantitative results for the total biomass of the *C. albicans* biofilm on crystal violet-stained samples after 1, 3, 5, and 7 days of microbial exposure, measured using the spectrophotometric method, are shown in Figure 3. On day 1, Groups 1 and 2 exhibited significantly lower OD values for the *C. albicans* biofilm compared to the control group (*p*  < 0.05); however, no significant difference was observed between these two experimental groups (*p*  > 0.05). On day 3, Group 2 demonstrated a significant reduction in the OD value of the biofilm compared to the control group (*p*  < 0.05), whereas no significant difference was noted between Group 1 and the control group (*p*  > 0.05). By days 5 and 7, the differences in OD values among the study groups were no longer statistically significant (*p*  > 0.05).

**Figure 4:**
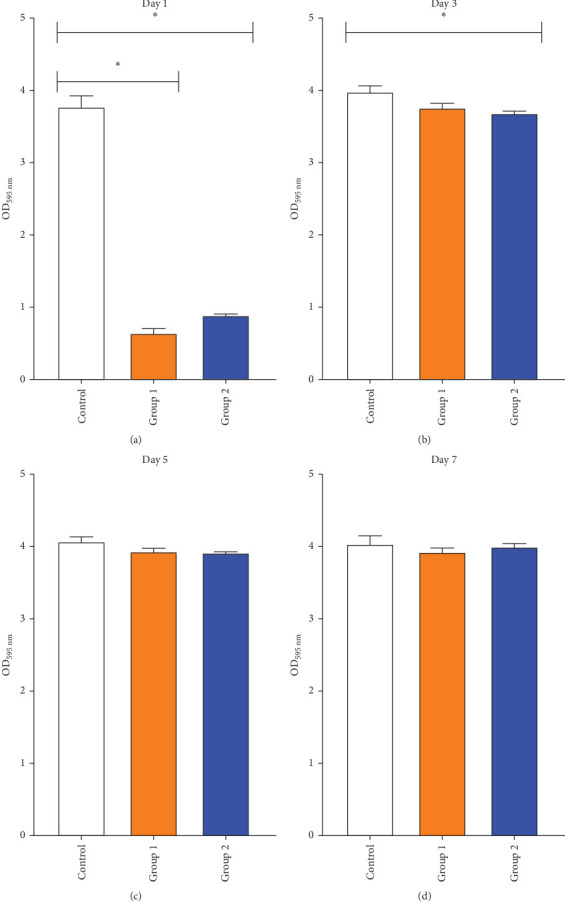
Inhibitory effect of tissue conditioners containing *O. gratissimum* EO on *S. mutans* biofilm formation at four time points postincubation. (A) Day 1; (B) Day 3; (C) Day 5; (D) Day 7, *⁣*^*∗*^*p* < 0.05 indicates statistical significance.

Figure 4 presents the quantitative analysis of the total biomass of the *S. mutans* biofilm on crystal violet-stained samples after 1, 3, 5, and 7 days of microbial exposure, as measured by the spectrophotometric method. On day 1, Groups 1 and 2 displayed significantly lower OD values for the *S. mutans* biofilm compared to the control group (*p*  < 0.05), with no notable difference between the two experimental groups (*p*  > 0.05). By day 3, Group 2 showed a significant decrease in OD value compared to the control group (*p*  < 0.05), whereas Group 1 did not vary significantly from the control (*p*  > 0.05). On days 5 and 7, the OD values across all study groups showed no statistically significant differences (*p*  > 0.05).

## 4. Discussion

The findings of this study resulted in dismissing the null hypothesis, which proposed that the inclusion of *O. gratissimum* EO would not affect the TBS or biofilm-inhibitory antimicrobial properties of the dental tissue conditioner. To the best of the author's knowledge, no previous research has explored the combination of *O. gratissimum* EO with tissue conditioners. However, studies have investigated the effects of other oils, such as coconut oil and oregano oil, on TBS and antimicrobial properties when incorporated into tissue conditioners [17, 18]. These studies reported that such oils either reduced or had no significant effect on TBS [17, 18], contrasting with our findings that tissue conditioners incorporating *O. gratissimum* EO enhanced TBS on day 1. In 2013, Srivastava et al. [17] reported that the addition of oregano oil to the tissue conditioner Visco-gel did not significantly affect TBS on days 1 and 7. Similarly, in 2017, Rawat et al. [18] found that oregano oil did not significantly affect TBS at 1, 3, or 7 days. In contrast, coconut oil was observed to reduce TBS after 24 h [18].

Acrylic polymer is an amorphous material that allows certain low-molecular-weight liquids to penetrate and diffuse, which can lead to cracking, plasticization, hardening, leaching, or dissolution of the polymer and its additives [19]. This effect depends on various factors, including the miscibility or solubility of the solvent [19]. For dental tissue conditioners, ethanol, and aromatic esters are two low-molecular-weight liquids commonly used as solvents and plasticizers [9]. The plasticizer lowers the glass transition temperature of the polymer below the oral temperature, ensuring the material remains in a soft state [20]. Ethanol swells the PMMA network of the acrylic denture base, allowing the plasticizer in the tissue conditioner to penetrate the swollen particles and create a bond between the two material layers [9].

Carvacrol and thymol, derivatives of aromatic alcohols found in oregano oil, have low polarity and are highly soluble in alcohol and organic solvents [17]. The similarity in solubility between the components of oregano oil and the plasticizers in tissue conditioners likely explains why mixing oregano oil with the tissue conditioner liquid component acts as a plasticizer and does not significantly alter TBS [17]. In contrast, the saturated fatty acids in coconut oil may undergo esterification reactions with ethanol in the tissue conditioner [18]. This interaction likely reduces the solvent's efficacy in the tissue conditioner, thereby negatively impacting TBS [18].

The *O. gratissimum* EO used in this study had eugenol as its primary volatile compound [16]. Eugenol, as a naturally derived compound, is widely used as a base material in various polymers due to its favorable chemical structure [21]. In this study, we hypothesized that eugenol integrates into both the polyethyl methacrylate chains in the tissue conditioner and the PMMA in the denture base resin, contributing to the increased TBS.

The *O. gratissimum* EO increased the TBS of the tissue conditioner to the denture base acrylic resin on the first day. However, this effect diminished by day 7, with no statistically significant difference in TBS noted between the experimental and control groups. This finding aligns with previous studies by Srivastava et al. [17] and Rawat et al. [18], which demonstrated that oregano oil and coconut oil did not affect the TBS between the two material surfaces at day 7. Thus, all three plant oils (*O. gratissimum*, oregano, and coconut) exhibited low stability in tissue conditioners and were highly susceptible to leaching into the environment.

During extended use in the oral cavity, tissue conditioners are prone to microbial buildup and colonization [22]. Our results showed that tissue conditioner incorporated with *O. gratissimum* EO effectively inhibited the biofilm formation of *C. albicans* in a dose-dependent manner on day 1 and 3. Similarly, in 2021, Hejazi et al. [12] reported that tissue conditioners combined with *Carum copticum* EO exhibited antimicrobial properties, with a significant reduction in absorbance readings at an EO concentration of 64 μg/mL compared to lower concentrations.

The antifungal property of tissue conditioner incorporating *O. gratissimum* EO gradually decreased over time, likely due to the leaching of EO and plasticizer components into the environment [12]. The decrease in plasticizer results in the hardening of the tissue conditioner and an increase in porosity [9]. Consequently, the relining layer needs to be replaced every 3–7 days [9].

*S. mutans* is a gram-positive spherical bacterium typically present in the oral cavity [23]. It plays a key role in the initiation of dental caries in both children and adults [23]. However, its involvement in the pathogenesis of oral mucositis remains controversial [24]. Some studies suggested that *S. mutans* formed a symbiotic relationship with *C. albicans*, enhancing the virulence of polymicrobial biofilms in a synergistic manner [25, 26]. Our results demonstrated that tissue conditioners incorporating *O. gratissimum* EO exhibited biofilm-inhibitory antibacterial efficacy against *S. mutans* in a dose-dependent manner, similar to their antifungal effect against *C. albicans*.

The limitations of this study include the lack of assessment of the long-term biocompatibility and safety of *O. gratissimum* EO in the oral environment, as well as its effectiveness against clinically isolated microbial strains. Despite these limitations, the study underscores the potential of *O. gratissimum* EO as an additive in tissue conditioners to mitigate the risk of infection, particularly during the healing process following surgery. Further investigations, including assessments of biocompatibility and cytotoxic activity, should be conducted before introducing *O. gratissimum* EO-incorporated tissue conditioners for clinical application.

## 5. Conclusion

Tissue conditioners containing *O. gratissimum* EO demonstrated a dose-dependent reduction in biofilm formation by *C. albicans* and *S. mutans* on days 1 and 3. Given that tissue conditioners are generally replaced every 3–7 days, incorporating *O. gratissimum* EO as an additive may help minimize biofilm development by these microorganisms without adversely affecting the TBS between the tissue conditioner and the denture base acrylic resin.

## Figures and Tables

**Table 1:** Composition of the tissue conditioner specimens tested with incorporated *O. gratissimum* EO.

Group	Tissue conditioner		*O. gratissimum* pure EO (μL)
	Powder (g)	Liquid (μL)	
Control	3.8	2500	0
Group 1 (1% EO)	3.8	2475	25
Group 2 (2% EO)	3.8	2450	50

Abbreviation: EO, essential oil.

## Data Availability

The data that support the findings of this study are available from the corresponding author upon reasonable request.
